# Peculiar Presentation of an Intrapericardial Ectopic Thyroid

**DOI:** 10.3390/reports9020127

**Published:** 2026-04-21

**Authors:** Stefano Auriemma, Riccardo Gherli, Lorenzo Giacometti, Annalisa Roveta, Pietro Rinaldi

**Affiliations:** 1 Department for Internal Medicine and Medical Therapy, University of Pavia, 27100 Pavia, Italy; 2 Cardiac Surgery Division, SS Antonio Biagio e Cesare Arrigo Hospital, 15121 Alessandria, Italy; r.gherli@hotmail.com; 3 Pathological Anatomy Division, SS Antonio Biagio e Cesare Arrigo Hospital, 15121 Alessandria, Italy; 4 Research and Innovation Department, SS Antonio Biagio e Cesare Arrigo Hospital, 15121 Alessandria, Italy; aroveta@ospedale.al.it; 5 Thoracic Surgery Division, SS Antonio Biagio e Cesare Arrigo Hospital, 15121 Alessandria, Italy

**Keywords:** ectopic thyroid, epicardial fat, mediastinal mass, ST-segment changes, ministernotomy, paraganglioma mimicry

## Abstract

**Background and Clinical Significance:** Intrapericardial ectopic thyroid tissue is extremely rare and can mimic vascular mediastinal or cardiac lesions. **Case Presentation:** We describe a 62-year-old woman with dyspnea, palpitations, and flushing for several months, progressively worsening, associated with nonspecific ST-segment abnormalities on ECG. Contrast-enhanced CT revealed a small, highly vascularized epicardial mass anterior to the ascending aorta. 18F-FDG PET/TC findings were inconclusive, and biopsy was not feasible due to the anatomical location. Surgical excision via upper ministernotomy was performed, leading to resolution of symptoms. Histology confirmed benign ectopic thyroid tissue. **Conclusions:** With fewer than ten similar intrapericardial cases reported in the English-language medical literature, this presentation underlines the diagnostic difficulty of such lesions and the importance of including ectopic thyroid tissue among the less common differential diagnostic considerations for intrapericardial masses, particularly in patients with prior thyroid disease.

## 1. Introduction and Clinical Significance

Ectopic thyroid tissue is a rare congenital condition resulting from abnormal migration of the thyroid anlage. It occurs more frequently in women and has an estimated prevalence of 1:100,000–300,000 in the general population. It is most commonly located along the thyroglossal tract—from the base of the tongue to the anterior neck—while mediastinal and intrathoracic localizations account for a small minority of cases. Reported prevalence is higher among patients with thyroid disorders, ranging from approximately 1:4000 to 1:8000 [1]. Intrapericardial ectopic thyroid tissue is rare, and localization within epicardial fat is even more unusual, with only few cases reported [2,3,4,5,6]. Reported lesions are frequently adjacent to the ascending aorta or supplied by coronary branches [3,4,5,6]. This entity presents a diagnostic challenge because it may clinically and radiologically mimic other intrathoracic or cardiac masses, including lipoma, pericardial cysts, vascular lesions, or neuroendocrine tumors. True ectopic thyroid tissue has no anatomical continuity with the orthotopic gland, and although the term “ectopic goiter” is sometimes used, it is not strictly accurate. In the diagnostic workup of suspected ectopic thyroid tissue, several imaging modalities may be employed depending on the clinical context. These include ultrasound for cervical evaluation, CT scan for anatomical and vascular relationships, MRI for soft-tissue and spatial assessment, nuclear medicine techniques such as thyroid scintigraphy, and 18F-FDG PET/TC only in selected cases [1]. Radionuclide thyroid scintigraphy was not performed in the present case because the lesion was not initially suspected to represent thyroid tissue. MRI was not requested due to the relative certainty regarding the lesion’s relationship with the surrounding structures obtained through the contrast CT-scan previously performed.

## 2. Case Presentation

**Figure 1:**
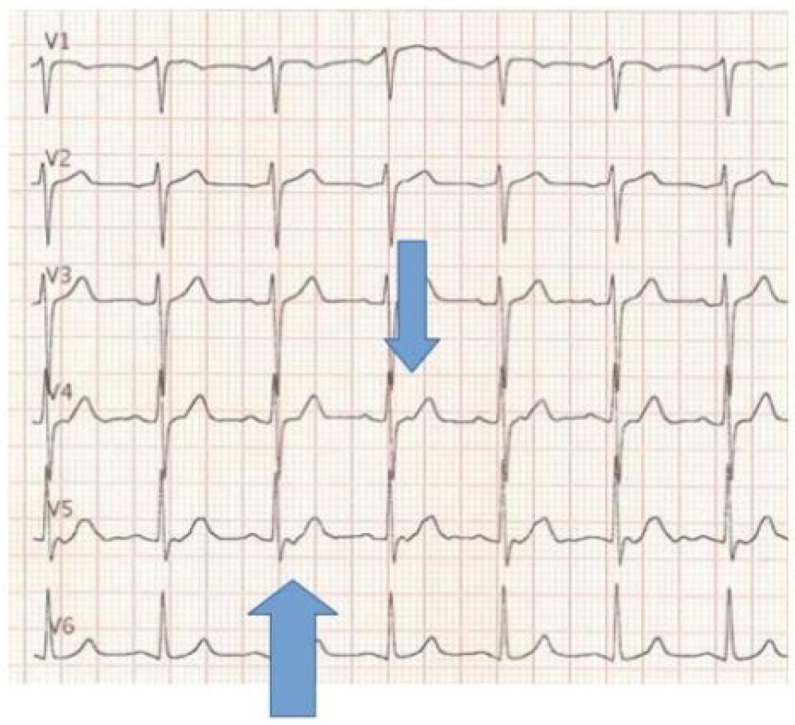
ECG showed non-specific ST-segments alterations in V4–V5.

A 62-year-old woman presented to the Emergency Department with dyspnea of several months’ duration, progressively worsening. She also reported associated symptoms as recurrent palpitations, tachycardia and skin flushing. Her only relevant past medical history was thyroidectomy for hyperthyroidism performed 16 years earlier. ECG revealed nonspecific ST-segment abnormalities in leads V4 and V5 [Figure 1].

**Figure 2:**
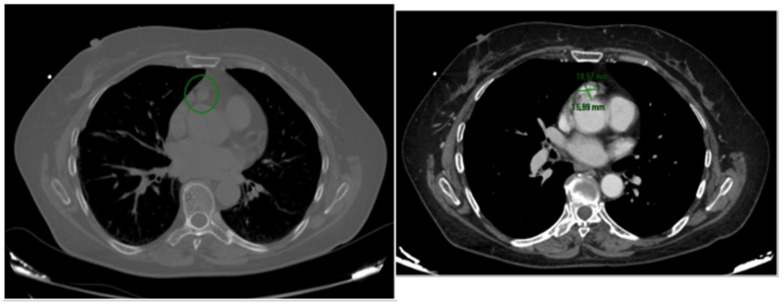
A CT scan with contrast was ordered.

Routine laboratory testing, including thyroid function tests and cardiac biomarkers, was unremarkable. Chest X-ray and contrast-enhanced CT demonstrated a 1.8 × 1.6 cm lesion within the epicardial fat anterior to the ascending aorta, located approximately 3 cm from the aortic valve. The mass showed a close relationship with the pericardium without evidence of invasion of adjacent structures. Proximity to the right coronary artery ostium was also noted [Figure 2].

Marked contrast enhancement indicated high vascularity, and heterogeneous enhancement with small low-attenuation areas was suggestive of focal necrosis. 18F-FDG PET/TC was subsequently performed and showed minimal, non-significant tracer uptake. MRI was not performed because the combination of contrast-enhanced CT findings, vascular pattern, anatomical location, and biochemical data led to a working diagnosis strongly suggestive of paraganglioma, and further investigations were not deemed necessary. A repeat CT scan obtained six months later demonstrated dimensional stability.

**Figure 3:**
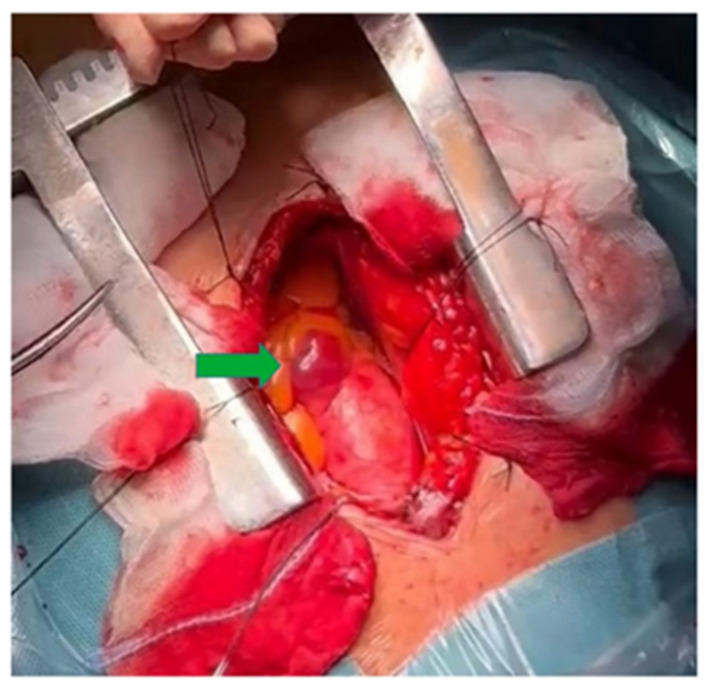
An upper ministernotomy was performed.

**Figure 4:**
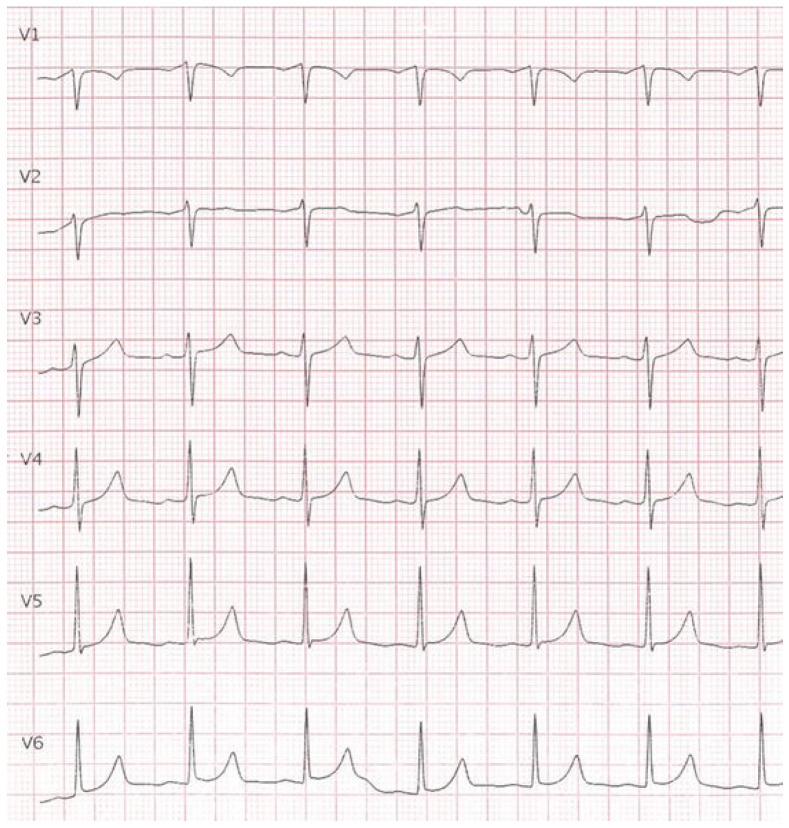
Post-op ECG shows absence of the alterations previously noted.

The anatomical position precluded CT-guided biopsy. Biochemical evaluation included urinary vanillylmandelic acid, which was above the reference range. Based on lesion location and biochemical findings, a periaortic paraganglioma was suspected. In cases where imaging is not definitive, biochemical findings suggestive of paraganglioma should not be taken as pathognomonic. Surgical excision was indicated, as it is commonly recommended for selected indeterminate mediastinal masses when malignancy cannot be excluded [7]. A median longitudinal upper ministernotomy was performed to minimize surgical invasiveness [Figure 3]. The postoperative course was uneventful, and the patient was discharged on postoperative day five. Follow-up ECG demonstrated resolution of the previously observed abnormalities, and all symptoms resolved [Figure 4].

**Figure 5:**
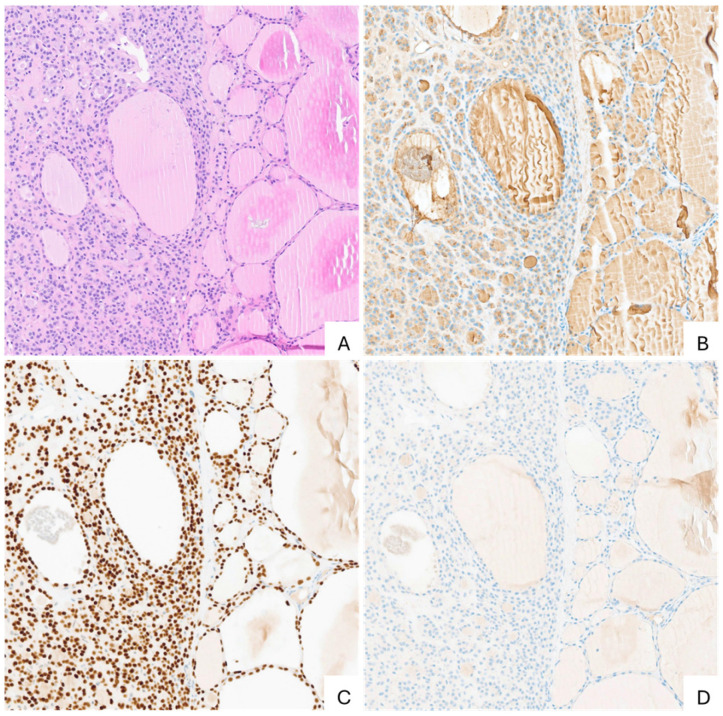
(**A**) Hematoxylin and eosin (40×). (**B**) Diffusely positive thyroglobulin immunohistochemical stain (40×). (**C**) Diffusely positive TTF-1 immunohistochemical stain (40×). (**D**) Negative cromogranine A immunohistochemical stain (40×).

Histopathological examination demonstrated thyroid tissue with diffuse thyroglobulin and TTF-1 expression and negative staining for chromogranin A and synaptophysin, excluding a neuroendocrine tumor [Figure 5].

## 3. Discussion

Evaluation of hypervascular intrapericardial masses remains complex due to biochemical misdirection, the limitations of metabolic imaging specificity, and the technical contraindications to percutaneous biopsy. Flushing, palpitations, and dyspnea are commonly associated with catecholamine-secreting tumors, and the elevation of urinary vanillylmandelic acid reinforced the suspicion of paraganglioma. However, biochemical markers alone are not diagnostic and may lead to overestimation if interpreted without adequate clinical and radiological correlation. Contrast-enhanced CT was central to lesion detection and characterization, demonstrating a small, intensely vascularized mass within epicardial fat and clearly defining its relationships with the ascending aorta, pericardium, and coronary ostium. These anatomical features also explained the infeasibility of percutaneous biopsy and directly influenced surgical planning. TC-PET 18FDG was inconclusive, showing only minimal uptake. This is consistent with the known variability of 18F-FDG uptake in thyroid and thyroid-related lesions, which can range from absent to intense, thereby limiting specificity in this setting [8]. MRI is described as a useful adjunct for soft-tissue characterization and more precise anatomical assessment in mediastinal and ectopic thyroid lesions [1]. In the present case, MRI was not performed because CT findings and biochemical data were considered sufficient by clinicians to guide management and proceed with surgical treatment. Based on published English-language reports, fewer than ten cases of intrapericardial ectopic thyroid tissue have been described since the first report in 1986 [3]. Most reported lesions are located near the ascending aorta and may present with palpitations, dyspnea, or angina-like symptoms, although many are incidental findings [2,4,5,6]. ECG abnormalities have rarely been emphasized in prior reports. In this case, nonspecific ST-segment changes resolved after resection together with symptom resolution, suggesting a possible association, although causality cannot be established from a single observation. Surgical resection has been the preferred management strategy in most reported cases, serving both diagnostic and therapeutic purposes, particularly when malignancy cannot be excluded [2,4,5,6].

## 4. Conclusions

Intrapericardial ectopic thyroid tissue is a rare kind of a hypervascular epicardial mass and may mimic other entities, including neuroendocrine tumors. In patients presenting with nonspecific chest-related symptoms and ECG abnormalities, a structural lesion involving the heart or great vessels may be included among the less common differential diagnostic considerations. Biochemical markers such as vanillymandelic acid may contribute to the diagnostic workup but should not be considered definitive in isolation. In this case, contrast-enhanced CT was decisive for anatomical definition, whereas 18F-FDG PET/TC did not provide conclusive characterization. MRI may be helpful in selected cases when CT findings are not sufficient for anatomical and tissue assessment. In our case, MRI was not performed because contrast-enhanced CT showed a well-defined surgical plane. In patients with prior thyroid disease or thyroidectomy, ectopic thyroid tissue may be considered among the less common diagnostic possibilities when clinical and imaging findings are not fully explained by more frequent conditions. Surgical excision remains an effective diagnostic and therapeutic option when noninvasive evaluation is inconclusive and symptoms persist.

## Data Availability

The original data presented in the study are included in the article, further inquiries can be directed to the corresponding author.
